# Enhanced tumor-infiltrating lymphocytes (eTIL) for cellular therapy of patients with pancreatic cancer or glioblastoma

**DOI:** 10.1186/2051-1426-3-S2-P35

**Published:** 2015-11-04

**Authors:** Qingda Meng, Zhenjiang Liu, Markus Maeurer, Elena Rangelova, Aditya Ambati, Thomas Poiret, Lalit Rane, Shanshan Xie, Bartek Jiri, Caroline Verbeke, Oscar Persson, Matthias Löhr, Ralf Segersvärd, Ernest Dodoo

**Affiliations:** 1 Karolinska Institutet, Stockholm, Sweden

## Purpose

The generation of T lymphocytes with specific reactivity against autologous tumor cells is a prerequisite for effective targeted cellular therapies. We established a protocol for tumor infiltrating lymphocytes (TILs) cultures from small biopsies or surgically resected material obtained from patients with pancreatic ductal adenocarcinoma (PDAC) and patients with glioblastoma.

## Methods

Tumor specimens obtained from 17 patients with PDAC and 16 patients with glioma were cultured with TIL medium, containing IL-2, IL-15 and IL-21. Activity of TILs directed against shared tumor associated antigens (TAAs, mesothelin, survivin and NY-ESO-1, EGFRvIII) was detected by Intracellular staining (ICS). Cytotoxicity was measured using a Chromium-51 release assay and the specific activity of TILs against autologous tumor was measured by INF-γ production. TCRVb analysis was investigated by a multiplex antibody TCR Vb specific panel. TCR clonality was gauged by CDR3 region PCR analysis and subsequent sequencing. The T cell phenotype, as well as activation/exhaustion marker profile was tested by flow cytometry.

## Results

We could reliably expand TILs, that resided predominantly in the central memory population (CD45RA^-^, CCR7^+^), from 33/33 patients. We were able to expand about 1.5 x 10e9 TIL from small PDAC biopsies. TIL showed reactivity to common TAAs, i.e. to mesothelin (16/17 in PDAC, 8/16 in glioma), survivin (12/17 in PDAC, 6/16 in glioma) or to NY-ESO-1(11/17 in PDAC, 6/16 in glioma) defined by intracellular cytokine staining. TIL cultures exhibited preferential usage of Vβ families (e.g. some TIL showed 99.3% Vb13.2, 99.60% Vβ2, 97.00% Vβ5.1 in CD4^+^ or CD8^+^ TIL). A PDAC Vb13.2^+^, CD8^+^ TIL clone strongly recognized autologous tumor cells, defined by INF-γ production, which could be blocked by the anti-HLA class 1 antibody (W6/32). TIL from glioma patients exhibited up to 25% IFN-γ and TNF-α production directed against autologous tumor cells, defined by ICS. TILs from PDAC and glioma showed strong cytolytic functions directed against autologous tumor cells, i.e. up to 70% specific lysis at an effector/target ratio at 25/1 by chromium-51 release assay.

## Conclusions

We have optimized methods for generating pancreatic cancer and glioblastoma specific TIL cultures from small resected tumor specimens. Tumor specimens are currently sequenced to detect potential targets for anti-PDAC and anti-glioma directed T cell clones. A Phase I study to administer TIL for patients with pancreatic cancer or glioblastoma will start at Karolinska.

